# LIF Inhibits Proliferation of Esophageal Squamous Carcinoma Cells by Radiation Mediated Through JAK-STAT Signaling Pathway

**DOI:** 10.7150/jca.81222

**Published:** 2023-02-22

**Authors:** Hongtao Luo, Zhen Yang, Qiuning Zhang, Tingdong Li, Ruifeng Liu, Shuangwu Feng, Zhiqiang Liu, Shilong Sun, Junru Chen, Xiaohu Wang, Xiaoling Xie

**Affiliations:** 1 Institute of Modern Physics, Chinese Academy of Sciences, Lanzhou, China.; 2 School of Public Health, Gansu University of Chinese Medicine, Lanzhou, China.; 3 Lanzhou Heavy Ion Hospital, Lanzhou, China.; 4 Gansu Provincial Cancer Hospital, Lanzhou, China.; 5 The Central Laboratory of the First Hospital of Lanzhou University, Lanzhou, China.

**Keywords:** Carbon ion, Esophageal squamous cell carcinoma, Proteomics, LIF

## Abstract

**Background:** Esophagus cancer is a malignant tumor with a high incidence rate, and radiation is an important modality for esophageal cancer therapy. However, therapeutic failure in the treatment of ESCC is often attributed to an inherent radio-resistance of the tumor cells. This study discusses effect and mechanism of carbon ion exerts tumor-inhibiting proliferation via down-regulation of LIF in esophageal squamous cell carcinoma.

**Methods:** Colony formation, CCK8 and EdU assays were used to detect cell survival and proliferation after 0 and 2Gy carbon ion irradiation of ECA109 cells. Proteomics changes were probed in response to carbon ion irradiation using quantitative proteomics approach incorporating TMT isotope tags. Then, candidate genes were identified via bioinformatics analysis methods and microarray results were verified by real-time qPCR. Paired ESCC tumor tissues and adjacent non-tumor samples from 17 patients were collected and used for detecting expression by immunohistochemistry. Furthermore, small interfering RNA (siRNA) was transfected into ECA109 and KYSE150 cells and cell proliferation was analyzed by EdU assay. Flow cytometry and Western blot were performed to measure the and apoptosis and JAK-STAT3 protein expression level of ECA109 and KYSE150 cells combined drugs after siLIF transfection.

**Results:** When compared with the control (0Gy), Inhibition of ECA109 cell proliferation and clonogenic survival by 2 Gy carbon ions, radiation group screened 360 differentially expressed proteins, 156 of which were up-regulated and 144 were down-regulated. Downregulation of LIF expression by siRNA enhances apoptotic in the ECA109 and KYSE150 cells, significantly inhibited esophageal squamous cell carcinoma cells proliferation. In ESCC cells, the JAK/STAT3 signaling pathway is inhibited in a LIF-dependent manner, resulting in the expression of STAT3 downstream target genes. Carbon ions combined with siLIF inhibited cell proliferation more significantly. The inhibitory cell proliferation effect was more pronounced by the combined intervention of carbon ion irradiation with siLIF. LIF expression was 18.51±9.84 and 5.82±4.50 in 17 paired ESCC tissues and adjacent non-cancerous tissues, respectively. LIF protein expression was lower in ESCC than in the adjacent normal tissue.

**Conclusion:** The findings of this study reveal that Carbon ion knockdown was shown to downregulate LIF in ESCC cells. LIF is involved in ESCC proliferation and inhibited the ESCC cell proliferation by activating the STAT3 signaling pathways.

## Background

Cancer of the esophagus is one of the most commonly occurring malignant tumors^1^. China is a high-incidence area of esophageal cancer (EC), over 90% of EC cases are esophageal squamous cell carcinoma (ESCC)^2^. For patients with unresectable esophageal carcinoma, who decline surgery, or who are medically unfit for surgery, radiotherapy has become the primary treatment option, is carried out in more than 80% of EC cases^3^. Currently, the X-rays used for radiotherapy of EC are low LET rays and cause damage to double-stranded DNA. Biological effects on tumor tissue are limited by factors such as cell cycle and hypoxia, which are unfavorable factors for the treatment of EC^4^. A high-LET carbon ion causes highly complex DNA lesions that include double-strand breaks. It has the advantages: it has not dependence on the oxygen concentration is small and killing effect is not affected by cell cycle^5^-^7^. It has a good therapeutic effect on radiation-insensitive or radioresistant tumors^8^, ^9^. Takahashi et al showed that carbon ion radiation had a significant inhibitory effect on tumor growth compared with X-rays. At the same time, carbon ion rays induced significant necrosis in the central region of the tumor, but X-ray induced only mild necrosis^10^. We have previously found that carbon ion radiation promotes apoptosis and inhibits cell proliferation in esophageal squamous carcinoma cells through cell experiments^11^. However, high LET radiation causes more complex protein modification changes through posttranslational and oxidative processes, making the specific targets and mechanisms of radiation-mediated biological phenotypes remain unclear. Mass spectrometry-based proteomic analysis can provide new and effective biomarkers for the early diagnosis of EC or predicting the prognosis of patients. LIF is a pleiotropic cytokine, was highly expressed on most solid tumors including nasopharyngeal carcinoma, pancreatic cancer and esophageal squamous cell carcinoma. In the development and occurrence of tumors, it plays an important role^12^. LIF has been shown to act as a carcinogenic mediator, playing an important role in tumor growth, proliferation and metastasis, and is associated with resistance to radiation therapy and chemotherapy^13^, ^14^. LIF, as an upstream protein molecule of STAT3 signaling pathway, may have an important role in the activation of STAT3 signaling pathway^15^. Existing research suggests that LIF signaling promotes human ESCC progression via SFK-dependent YAP activation and is a new potential target of treatment for human ESCC^16^. This study investigated LIF's effects on cell proliferation and apoptosis after carbon ion irradiation of esophageal squamous carcinoma cells, as well as its potential molecular mechanisms.

## Materials and Methods

### Patients and samples

Cancer tissues and para-cancerous tissues of patients were collected from Tumor Hospital of Gansu Province. Tumor and para-cancerous tissues were obtained from 17 patients with ESCC, who had surgery to resect the tumor between January 2017 to January 2018. The inclusion criteria were as follows: (a) histological proof of thoracic ESCC; (b) complete surgical resection (R0); (c) no neoadjuvant or adjuvant treatment. Para-cancerous tissues 2 cm away from the tumors were used as control.

### Reagents

The EdU kits were purchased from Guangzhou RiboBio Co., Ltd. Meanwhile, siRNA LIF and Shanghai Jima Biotechnology Co., Ltd. synthesized siRNAs LIF and negative control. TMT (Tandem Mass TagTM) kits were purchased from Thermo Fisher Scientific (NJ, USA). The following antibodies were used for western blotting: anti- STAT3 (Cat. No. 12640, CST), anti-LIF (Cat.No.26757-1-AP), anti-JAK1 (Cat.No.66466-1-Ig) and horseradish peroxidase-conjugated secondary antibodies (Cat. No. 150159) were from Proteintech (Wuhan, China).

### Cell culture

ECA109 and KYSE150 were purchased from Cell Bank, Shanghai Institutes for Biological Sciences, Chinese Academy of Sciences (Shanghai, China). The cells were maintained in RIPM-1640 medium containing 10% fetal bovine serum (FBS), 100 U/mL penicillin and 0.1 mg/mL streptomycin. Cells were cultured at 37°C with 5% CO2 and 95% humidity in an incubator.

### Irradiation conditions

A carbon ion beam (field, 10 cm × 10 cm, Bragg peaks, 5mm, dose rate, 1 Gy/min) of 100 MeV/u was supplied by the Heavy Ion Research Facility in Lanzhou (HIRFL) at the Institute of Modern Physics, Chinese Academy of Sciences (IMP-CAS). After being exposed to 2Gy of carbon ion radiation, the cells were harvested. Three independent replicates were included in each experiment.

### 2.5 Cell proliferation and clonogenic survival assays

Cells were seeded into six-well plates with200, 1000 cells/well in 2ml culture medium, adherent cells were irradiated with carbon ions and the cells were cultured in 5% CO2 incubator at 37 °C. When the number of clones exceeded 50, the supernatant was discarded, and the cells were fixed with 4% paraformaldehyde for 15 min at room temperature. Then all plates were stained with a 0.1% crystal violet solution for 5 min and rinsed under running water. Colony formation rate of the irradiated group divided by colony formation rate of the control group was calculated as the survival fraction (SF). ECA109 Cells were placed at a density of 3000 cells per well in 100μl medium in 96‐well plates, and the cells were exposed to carbon ions. At 24, 48, 72, and 96 h, 10 μl of CCK-8 solution was added into each well and incubated for another 2 h. Absorbance at 450 nM (A450) was read on a microplate reader, and proliferation curves were plotted.

Ethynyl deoxyuridine (EdU) incorporation assay was performed to assess cells proliferation. Briefly, at 48 h after intervened with carbon ions, cells were harvested and reseeded in 48-well plates for EdU assays, respectively. The rate of cell proliferation was measured using an EdU cell proliferation assay kit (RiBoBio, China), according to the manufacturer's protocol.

### Quantitative proteomics analysis of TMT-labeled

The samples were supplemented with SDT lysis buffer (4% SDS, 100 mM Tris-HCl, 1 mM DTT, pH 7.6) and then boiled for 15 min. The cell lysate was centrifuged at 14000g for 15min at 4℃ to recover the supernatant. Proteins were quantified by the BCA method. Immediately, 20 µg proteins were mixed with 5 × loading buffer (Beyotime) to heat in boiling water bath for 10min to produce the protein samples. Samples were analyzed by 12% SDS-PAGE and Coomassie Brilliant blue staining. Totals protein was extracted and subsequent FASP digestion was performed. Samples were centrifuged at high speed for 10mins. In each group, 100μg peptide mixture of each sample was labeled using TMT reagent according to the manufacturer's instructions (Thermo Fisher Scientific). Samples were separated by HPLC using an Easy-nLC system with nanoliter flow rate. They were then analyzed using an EASY-nLC 1200 (Thermo Fisher) coupled to a QC-Exactive Plus mass spectrometer (Thermo Fisher).

### Cell apoptosis assay

We performed a double staining with annexin V-FITC/PI (BD) to confirm that carbon ions cause cell death in A549 cells. We harvested the cells, washed them twice with cold PBS, resuspended them in 1× binding buffer, and stained them with 5μl Annexin V-FITC and 5μl PI as directed by the manufacturer. Finally, the samples were analyzed by a flow cytometer using CytExpert software.

### Immunohistochemical staining

Fixing the tissues with 4% paraformaldehyde and embedding them in paraffin, sections were cut at 4μm thickness and immunohistochemical staining of LIF performed with corresponding antibodies. The degree of staining was determined based on the percentage of positive cells was categorized into the following four grades: blue is negative, light yellow as weak positive, brown-yellow is medium positive and dark brown is strongly positive. The IHC staining indices was calculated as the mean gray values of positive cells (staining intensity) and the percentage of positive-staining area (staining area).

### qPCR and Western blotting

The cells were lysed and mRNA was extracted by TRIzol (Invitrogen) according to the manufacturer's instructions. Then, the RNA samples were used for complementary DNA synthesis using the reverse transcription kit Hifair TM II 1st Strand cDNA Synthesis Supermix kit with gDNA Eraser (YEASEN, Shanghai, China). Next, Using the primers, Hieff's qPCR SYBR Green Master Mix kit (Yeasen, Shanghai, China) was used to perform real-time quantitative PCR. DNA amplification by PCR was performed using a T100 thermal cycler (Bio-Rad, CA, USA). Data were expressed as a fold change of mRNA after following calculation ∆∆Ct = mean ∆Ct value (target samples) - mean ∆Ct value (control samples), where fold change value corresponds to the 2 ^-∆∆Ct^, The ACTB expression level was measured as the internal control to normalize the data. Standard western blot methods were used. The cells were collected and their total protein was extracted. Then, the total protein concentration was then determined by the BCA protein assay reagent kit, and the cell lysates were separated on SDS-PAGE using 12% gels and transferred to nitrocellulose membranes. The membranes were blocked with 5% skimmed milk in PBS-0.1% Tween 20. The membranes were incubated with the primary antibodies overnight at 4°C, followed by incubation with the corresponding secondary antibodies for 1 h at room temperature. We analyzed the signal by densitometry using a ChemiDocXRS+ imaging system and ImageLab software from Bio-Rad.

### Data Analysis

For the evaluation of data quality, false positive has been removed through protein FDR≤0.01, site FDR≤0.01, and other standards during the database search. The acquired MS/MS data were analyzed against UniProt Homo sapiens database (http://www.uniprot.org/).The protein quantitation data results were analyzed using the Mann-Whitney test, defined as p < 0.05 and |log2FC| > *(ratio > * or ratio < * [fold change, FC]), were used to screen the differentially expressed proteins (DEP).Gene ontology enrichment and KEGG pathway analyses GO annotation and KEGG enrichment analyses were conducted to annotate the potential function of the genes. The experiments were conducted in triplicate unless otherwise specified. Data were presented as a mean ± SD, with statistical analysis performed using GraphPad Prism 7. When only two groups were compared, Student's t test was used.

## Results

### Carbon ions inhibits ESCC cells clonogenic survival and cell proliferation

**Figure 1:**
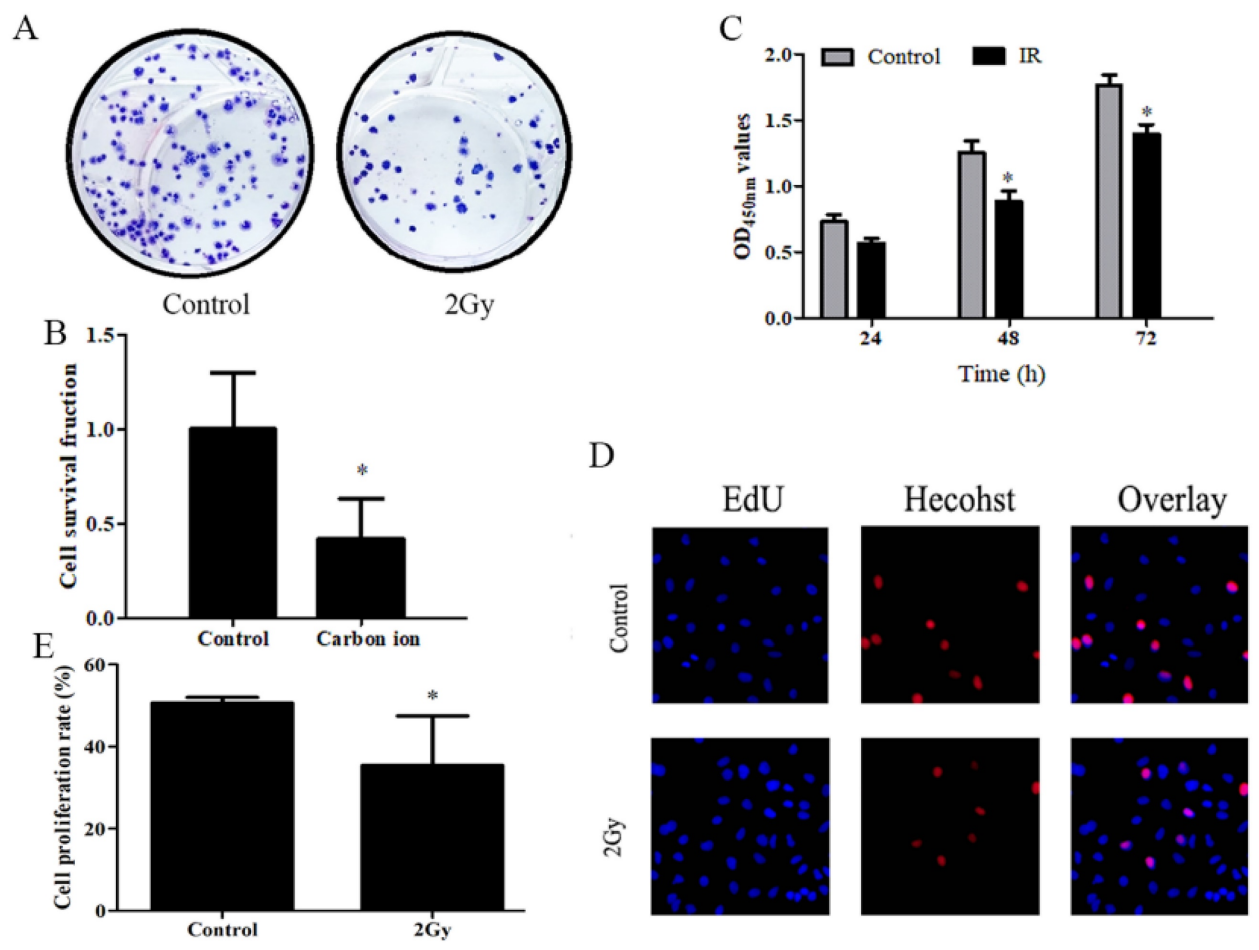
Carbon ion radiation inhibits the proliferation of ESCC cells. A. ECA109 cellular clone formation. B. Cell clonogenic ability was evaluated by cell survival fraction. C. CCK8 to detect cell proliferation change. D. EdU staining of ECA109 cells and EdU-positive cell proportion. E. Quantification of EdU-positive myocytes.

A study was conducted to determine whether carbon ions have any effect on the growth of ESCC cells, ESCC cells proliferation was assessed using CCK8 and clonogenic survival assay. After irradiation, cell proliferation was examined using CCK8 and clone formation assays. Following irradiation survival fraction of ECA109 cells after exposure to different dose of carbon ion radiation was shown in Figure 1. clonogenic survival assays finding on day 20 post irradiation (Figure 1A), compared to 0Gy controls (Figure 1B), the cloning ability of the ECA109 cells after 2Gy radiation were significantly decreased (p<0.05). The surviving fraction decreased ∼60% in the radiation (2Gy) compared to the controls clone. We performed CCK8 assay and found that 2Gy carbon ion remarkably decreased at different times after radiation proliferation abilities of ECA109 cells (Figure 1C). EdU proliferation assays showed a similar result (Figure 1D), cell proliferation was significantly inhibited 48h after treatment with 2Gy carbon ion when compared to those after 0Gy radiation (p <0.05 (Figure 1E).

### iTRAQ quantitative analysis of carbon ion induced proteomics changes

**Figure 2:**
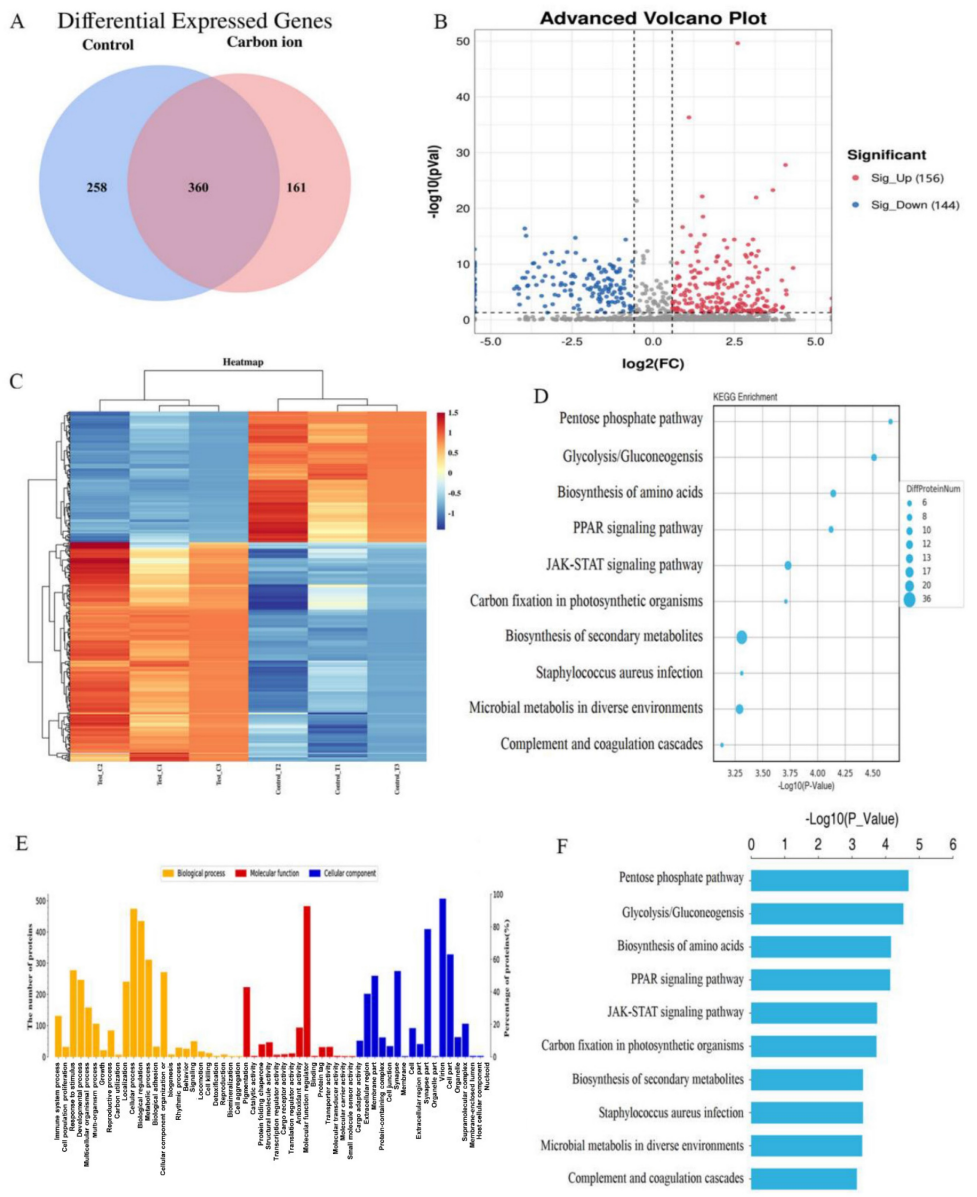
Comparative proteomic analysis of ECA109 cell with iTRAQ quantitative proteomics A. Venn diagram of differentially expressed proteins. B. Volcano plot depicting the analysis of the differentially expressed proteins. C. Heatmap representing hierarchical clustering of differentially expressed proteins. D. The KEGG Pathway analysis of the differentially expressed proteins. E. GO enrichment analysis of differential proteins. F. Histogram of GO enrichment of differentially expressed proteins.

After the analysis of the protein expression profiles of irradiation induce, 521 and 618 different molecules were identified in ECA109 cells after 0 and 2 Gy irradiation groups, respectively (Figure 2A). In above analysis, 360 overlaps existed were found between the two groups of differentially expressed proteins, with 156 upregulated and 144 downregulated proteins (Figure 2B). After the hierarchical clustering analysis, and was classified into one of three categories, red indicates up-regulation, blue indicates down-regulation (Figure 2C). To get a deeper understanding of the DEG molecules, we then performed KEGG and GO enrichment analysis (Figure 2D,E). These results showed that carbon ion radiation-induced protein factors can influence tumor cell biological behavior by multiple signaling pathway (Biosynthesis of amino acids, PPAR signaling pathway and JAK-STAT signaling pathway) (Figure 2F). We analyzed the data and selected the 20 differentially expressed protein in each cluster of populations (Table 1). Among them, 10 were upregulated (NUSAP1, A2M, DEGS1, AHR, LPCAT2, INCENP, KIF20A, SLC29A1, JAGN1, EXTL2) and 10 were downregulated (HMGCS1, HMGN3, LSM3, TKT, LIF, H3-3A, JPT1, NIBAN2, SLC9A3R1, KHSRP) (Table 1). Among differentially expressed cytokines, members of the IL-6 family (LIF) were noteworthy.

The binding of IL-6 to its receptor canonically activates the JAK-STAT3 signal pathway. Stat3 is a key mediator in gp130 signaling and a major target of JAKs. LIF, a pivotal gene involved in carcinogenesis, is as an important effector of the oncogene-driven pathways linking inflammation to cancer. Therefore, we can do further in-depth research on this.

### LIF expression differences in EC patients

**Figure 3:**
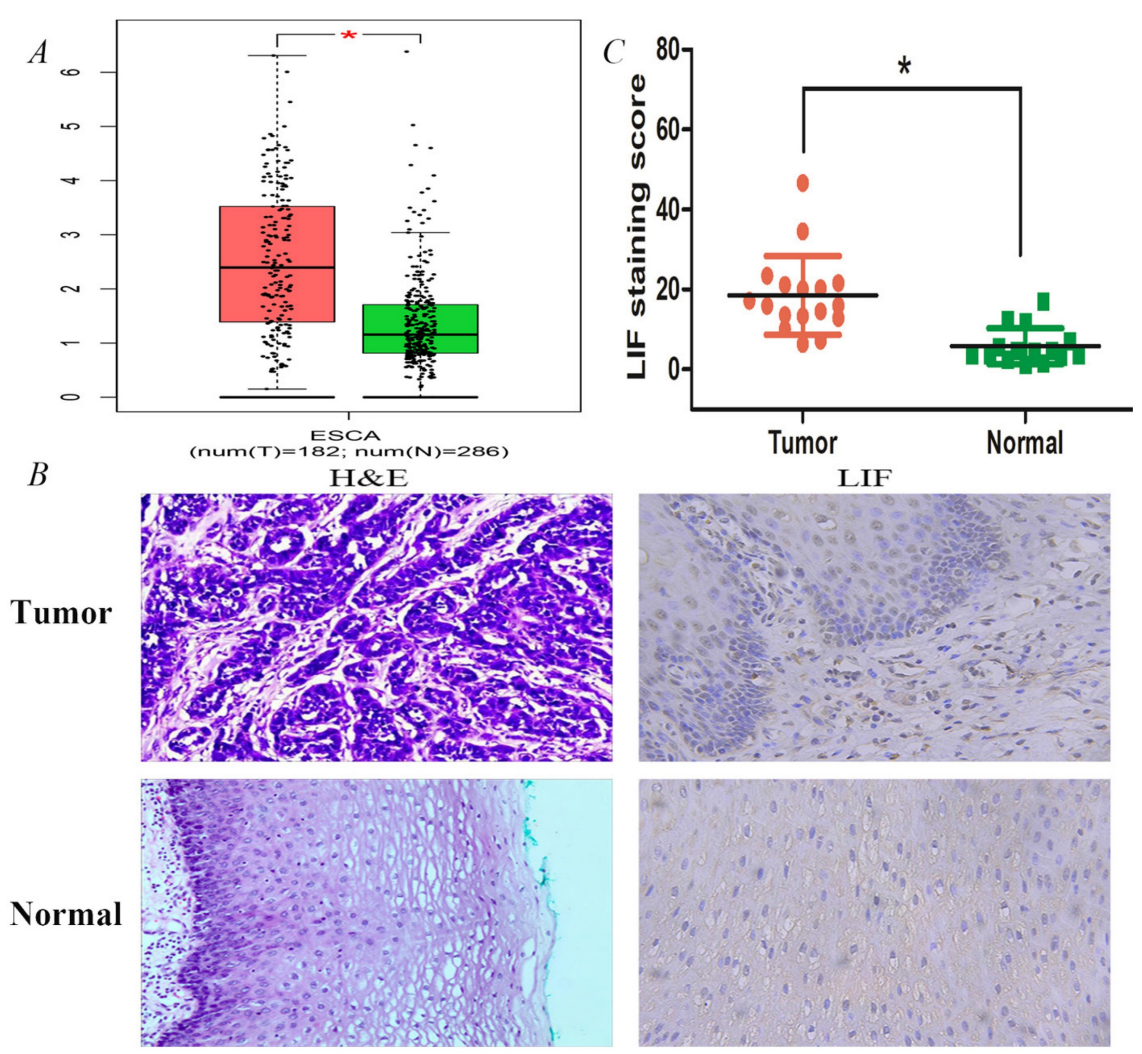
Expression validation of LIF using single molecule in databases and tissues samples. A. The expression differences of LIF between ESCA patients and normal controls were conducted by GEPIA dataset. B. HE staining and IHC staining of LIF in ESCC tumor and normal tissues. C. The LIF staining scores for the ESCC tissue samples were higher than those for their adjacent normal tissue samples.

LIF expression analysis was also performed in normal and ES tissues of the online GEPIA database (http://gepia.cancer-pku.cn/) (Figure 3). As shown in Figure 3A, the protein expression of LIF in EC tissues (n=182) was higher than that in normal tissues (n=286). Immunohistochemistry analysis was further performed to examine the expression of LIF in 17 human EC samples and corresponding para-cancerous esophageal tissues (Figure 3B). According to the results, tumor tissues expressed significantly more LIF than adjacent tissues, their correspond LIF expressions were 18.51 ± 9.84 and 5.82 ± 4.50 in 17, respectively (Figure 3C).

### LIF regulates proliferation of ECA109 and KYSE150 cells

**Figure 4:**
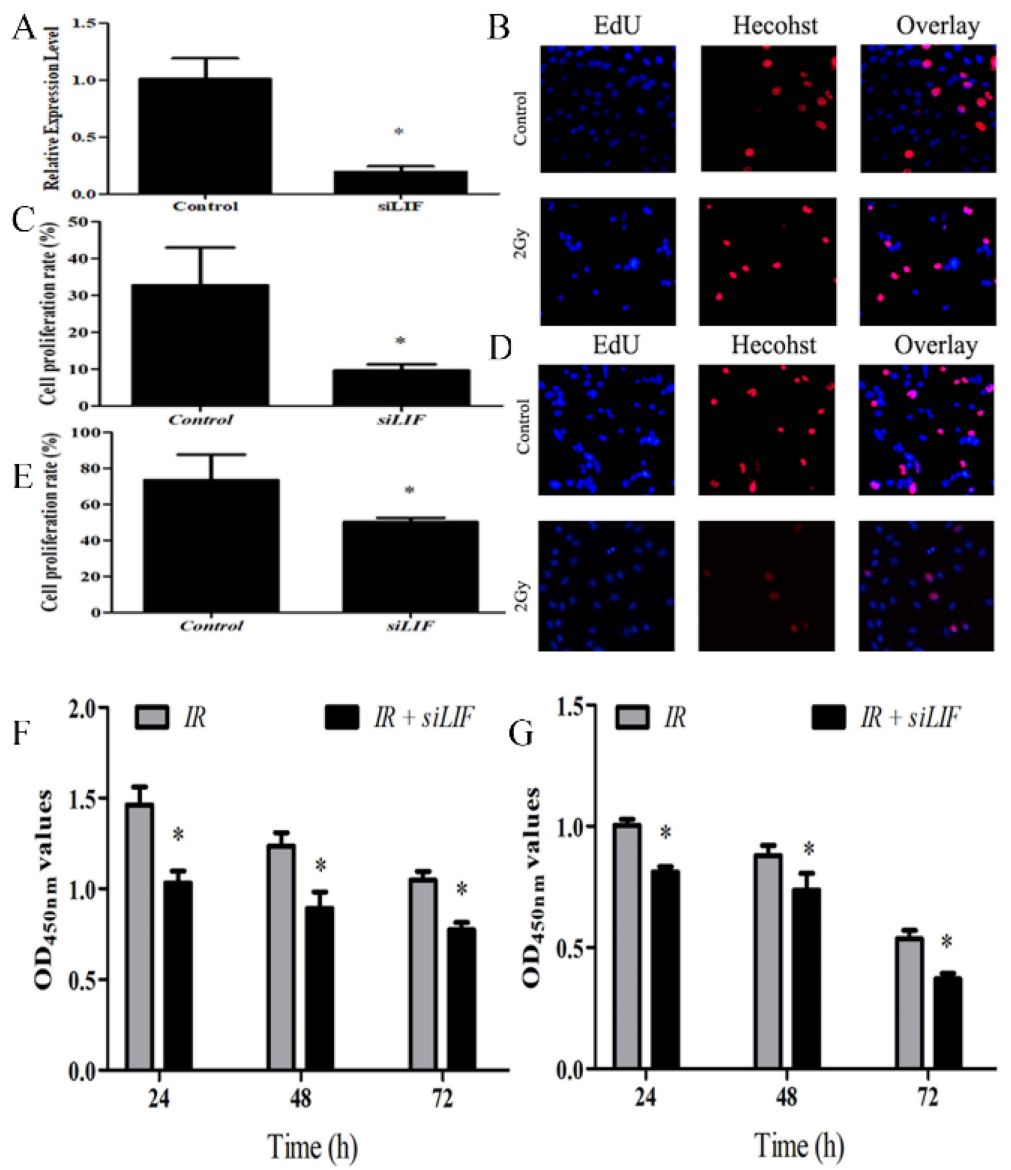
A. PCR analysis of transfection efficiency of siRNA. B. Cells were transfected with siRNA, and 48 h later, the cells were subjected to EdU incorporation assay. C. The percentage of EdU-positive cells (%) was calculated as the EdU-positive cell number/the total cell number in human ECA109 cells. D. EDU incorporation assay showed the EDU-positive cells in human KYSE150 cells after transfection of control or siLIF. E. Quantification of Edu positive KYSE150 cell number. F-G. ECA109 and KYSE150 cells transfected with siLIF were treated with carbon ion (2Gy) for 48 h, and cell proliferation was determined by CCK8 assay.

To evaluate the effects of LIF on tumor growth, ECA109 cells were transfected with siRNA segments against LIF to knockdown LIF expression (Fig 4). The efficiency of the siRNA down-regulating the expression of LIF mRNA was determined by real-time quantitative PCR analysis. The efficiency of gene silencing LIF was 81.05±4.11% (Figure 4A), indicating that the siRNA expression vector is an efficient method to downregulate LIF gene expression. Subsequently, the EdU assay was used to evaluate the proliferation of KYSE150 and ECA109 cells with LIF knockdown (Figure 4B, 4D). These results indicated that compared with the control group, the proliferation of the KYSE150 and ECA109 cells was significantly inhibited by transfection with LIF siRNA (Figure 4C, 4E). The cells were treated with carbon ion for 48 hours and CCK8 experiments found the similar results with EdU fluorescence experiments (Figure 4F, 4G) respectively.

### Low LIF expression induced apoptosis in ECA109 and KYSE150 cells

**Figure 5:**
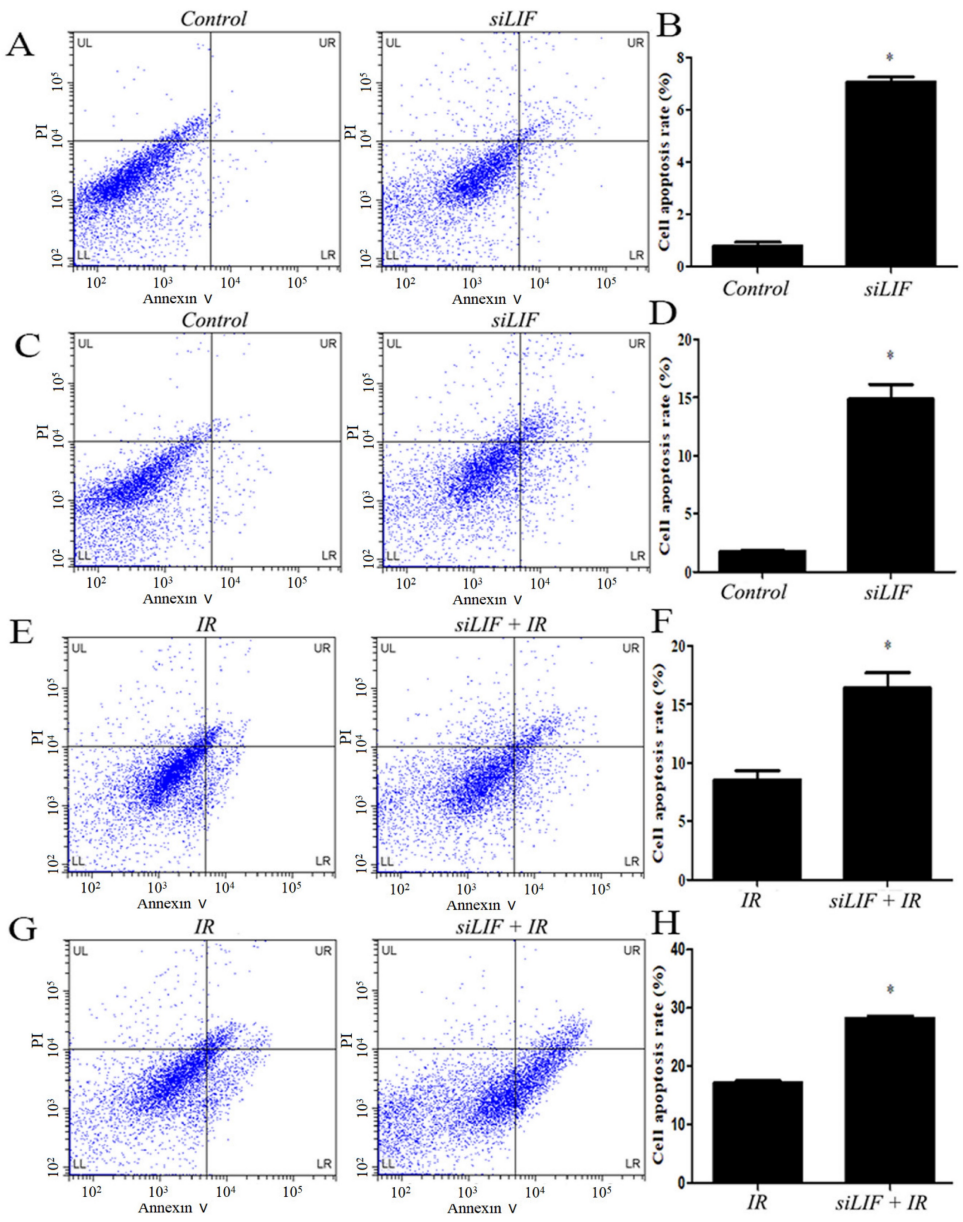
Induction of apoptosis after siRNA or carbon ion treatments. A. Flow cytomerty of ECA109 apoptotic cells with control or siRNA. B. ECA109 apoptotic cell numbers were significantly higher in siRNA groups. C. The KYSE150 cell apoptosis was detected by flow cytometry. D. LIF siRNA induced increase in the cell apoptosis percentage of KYSE150 cells. E. The ECA109 cell apoptosis was analyzed siRNA transfection by flow cytometry. F. The data of relative cell apoptosis in carbon ion and LIF siRNA group ECA109 cells. G. The KYSE150 cell apoptosis was analyzed siRNA transfection or combined with carbon ion(2Gy) by flow cytometry. H. There was KYSE150 cell apoptosis rate increase in LIF siRNA combined with carbon ion than single IR group.

Thereafter, the KYSE150 and ECA109 cells with LIF knockdown were harvested and examined for apoptosis by flow cytometry (Figure 5A, 5C). In ECA109 and KYSE150 cells, the percentage of apoptotic cells in siLIF groups was 7.07±0.19 and 14.85±1.28 and in control groups were 0.78±0.14 and 1.73±0.75, respectively (Figure 5B,5D). The results demonstrated that the LIF knockdown significantly induce apoptosis in KYSE150 and ECA109 cells. To gain further insight into the relationship of carbon ion radiation with LIF expression, after siRNA knockdown, cells were irradiated with 2Gy carbon ion (Figure 5E,5G). Apoptotic rates of ECA109, KYSE150 cells treated with LIF siRNA combined with carbon ion(2Gy) were 16.39±1.34, 28.28±0.41, whereas those in the radiation groups were 8.51±0.81 and 17.17±0.30, respectively. It was found that the combination of radiation and siLIF significantly increased KYSE150 and ECA109 cell apoptosis (Figure 5F,5H).

### LIF was the main regulator of proliferation of ESCC cytokine inducing the JAK-STAT3 pathway

**Figure 6:**
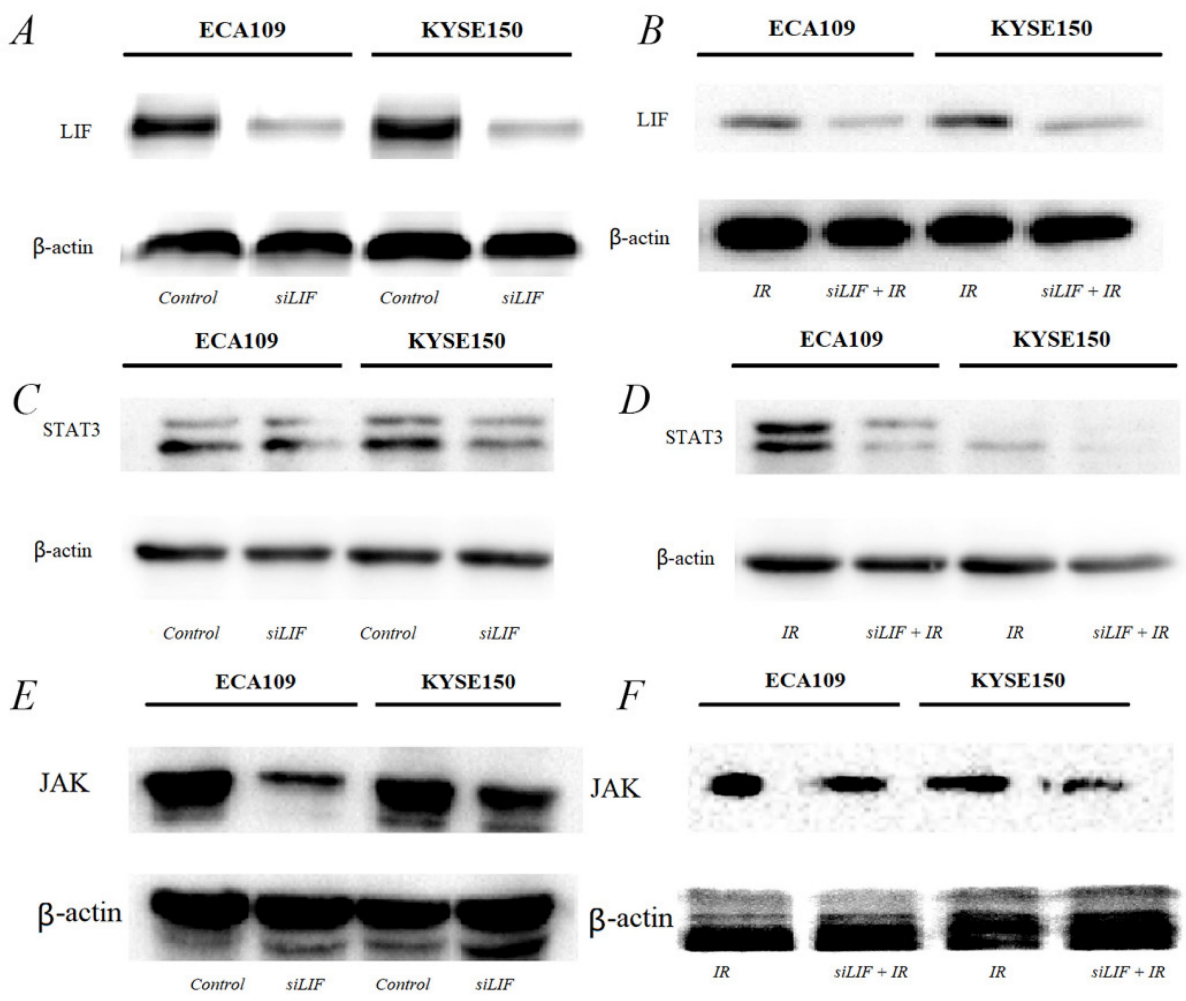
Western blot results for the expression of molecules in the JAK-STAT3 pathway. A, C, E. LIF, STAT3, JAK proteins were expressed in ECA109 and KYSE150 cells. B, D, F. LIF, STAT3, JAK proteins expression following transfection with siLIF/carbon ion combination irradiation in ECA109 and KYSE150 cells.

The results of the bioinformatics analysis that LIF regulate ESCC development and tumorigenesis by participating in the JAK-STAT3 signaling pathway. Next, we were also confirming that low-LIF expression could markedly inhibit JAK/STAT3 expression in both ESCC cellular models (Figure 6A, B). Western blot analysis indicated when LIF was silenced by siRNA, the expression of STAT3 and JAK were downregulated simultaneously (Figure 6C, D). STAT3 and JAK protein expression are even more obvious downregulated in both ESCC cellular models after carbon ion irradiation was combined with siLIF. This finding indicated that siLIF and carbon ion cooperatively regulate ESCC proliferation (Figure 6E,F).

## Discussion

The epidemic trend of EC varies greatly between China and the West, with a low incidence in Europe and the United State. The predominance of pathological type was ESCC. ESCC has a high prevalence in several countries in Africa and Asia. And esophageal squamous epithelioid cell carcinoma is the predominant pathological type^17^. Due to EC occult onset and the lack of typical clinical symptoms and signs in early stage. thus, most of the patients are already in middle or late periods while visiting doctors. The use of radiation therapy is an effective treatment for cancer patients who are medically inoperable or have surgically unresectable esophageal carcinoma. Despite remarkable advancement in X-ray radiotherapy equipment and modern accurate radiotherapy technologies, recent improvement of EC management strategies including radiotherapy, chemotherapy and target therapy has increased the therapeutic efficacy. However, the overall five-year survival of patients with esophageal carcinoma is remains lower than 40%^18^. Clinically, conventional radiation therapy for EC generally uses a single 1.8 to 2.0 Gy fractionated irradiation method. For EC patients treated with RT alone, the 5-year survival rate of EC was only approximately 8-16%. The main reasons for low efficacy were uncontrolled tumor and/or local recurrence^19^. A radiation-resistant tumor might benefit from carbon ion therapy because carbon ions have a higher LET and a higher biologic efficacy. It was reported that the carbon ion irradiation more effectively inhibits tumor growth, compared with X-ray irradiation. The RBE of the carbon ion was 2.02 relative to X-ray. Histopathology demonstrated that carbon ion (20 GyE) can cause marked necrosis in the central area of the tumor, only multinucleated giant cells and inflammatory cells in surrounding area. However, X-ray resulted in slight necrosis in the central area of the tumor, and tumor cell repopulation around the necrotic areas^10^. The ESCC for combined carbon ion and CHAP31 treatment, CHAP31 suppressed the expression of DNA repair-related genes and enhanced carbon ion radiosensitivity^20^. Our previous carbon ion irradiation studies of ESCC cells in different differentiation states found that a high dose of carbon ions prolongs G2/M cell cycle arrest, promotes apoptosis, and inhibits cell proliferation significantly. Carbon ion inhibiting sustained STAT3 activation via blocking of the JAK2/STAT3 signaling pathway, which leads to inhibition of migration and invasion in ESCC cells^11^.

Proteomics provides a powerful means of understanding cancer pathophysiology and identifying new therapeutic targets. Carbon ion radiation has been shown to induce transcription and translation of VEGF-alpha in cancer cells^21^. Based on these previous studies, further in-depth proteomic profiling analyses protein expression in ECA109 cells irradiated with 2 Gy carbon ions. From these, 360 proteins were found to be differentially expressed. Here, we focus on the LIF antitumor function. Then, GO enrichment and KEGG pathway analysis show that LIF participates in regulating several crucial processes and signaling pathways. Interestingly, LIF plays a role in ESCC cell proliferation through inhibiting the STAT3 signaling pathway. It was observed that several types of cancer express abnormal levels of LIF, which is associated with tumorigenesis, development, invasion and metastasis^15^, ^22^. LIF levels are increased in the serum of nasopharyngeal carcinoma patients, and positive correlation with poor treatment response and local tumor recurrence^13^. LIF levels have been found to be significantly elevated in the serum of patients with esophageal adenocarcinoma before treatment. This is consistent with our results that increased secretion of LIF protein is associated with radiation resistance^14^. LIF has been found to play a key role in the development of PDAC, and can be used as an effective therapeutic target and circulating marker, with great potential for medical translation^23^. In this study, the differential expression of LIF was analyzed in tissue samples and blood samples from patients with ESCC. The results showed that the expression of LIF in tissue and blood of patients with ESCC was significantly higher than that in adjacent normal tissues and blood of healthy subjects. Overexpression of LIF can induce G1 arrest of tumor cells and thus inhibit the proliferation of gastric cancer cells^24^. This study irradiated ECA109 cells with carbon ions, it was found that carbon ion rays could reduce LIF expression in esophageal squamous carcinoma cells, thereby inhibiting tumor cell proliferation. LIF, which is under-expressed in EC, significantly inhibits tumor cell proliferation, allowing increased carbon-ion radiation-induced sensitivity. Carbon ions have a greater lethality to tumor cells with low LIF expression and induce apoptosis in EC cells with low LIF expression.

The JAK/STAT3 signaling pathway is activated in a LIF-dependent manner, resulting in the expression of STAT3 downstream target genes^25^. STAT3 is associated with tumor cell proliferation, invasion and immunosuppression, and JAK-STAT3 signaling promotes cancer through inflammation^26^. Inhibit STAT3-mediated gene regulation, block tumor cell proliferation and selectively induce apoptosis of tumor cells with activated STAT3. Cell proliferation is regulated by the JAK2/STAT3 signaling pathway^27^. As STAT3 activation promotes tumorigenesis through its effects on cell proliferation, differentiation, and anti-apoptosis, the STAT3 signaling pathway is a potential target for tumor therapy^28^. In this study, we found that LIF regulated the biological behavior of tumors through the JAK-STAT3 signaling pathway by bioinformatics analysis, while we observed that low LIF expression decreased the expression of JAK and STAT3 factors. Therefore, in this study, we speculated that carbon ions inhibited the proliferation of EC cells by decreasing LIF expression and inhibiting STAT3 signaling pathway, which was further confirmed by siLIF experiments.

In summary, we used quantitative proteomics to screen differentially expressed molecules LIF in esophageal squamous carcinoma cells after carbon ion irradiation. The LIF expression were further validated in the clinical patients. In this study, we preliminarily revealed that carbon ions inhibited the proliferation of ESCC cells by regulating STAT3 signaling pathway through down-regulating LIF in ESCC cells.

## Figures and Tables

**Table 1:** Differential protein factor after exposure to 2 Gy carbon-ion beam radiation

No.	Accession	Name	MW [kDa]	Aver Control	Aver Carbon ion	FC	FDR
1	Q9BXS6	NUSAP1	49.4	80.35	128.85	0.6813	0.0409
2	P01023	A2M	163.00	82.05	131.15	0.6766	0.0425
3	O15121	DEGS1	37.80	78.85	125.9	0.6751	0.0430
4	P35869	AHR	96.10	78.45	125.2	0.6744	0.0430
5	Q7L5N7	LPCAT2	60.20	81.20	129.5	0.6734	0.0432
6	Q9NQS7	INCENP	105.00	84.50	134.65	0.6722	0.0435
7	O95235	KIF20A	100.00	83.35	132.4	0.6676	0.0454
8	Q99808	SLC29A1	50.20	86.10	136.6	0.6659	0.0459
9	Q8N5M9	JAGN1	21.10	82.55	130.9	0.6651	0.0460
10	Q9UBQ6	EXTL2	37.40	85.30	134.55	0.6575	0.0496
11	Q01581	HMGCS1	57.30	111.35	75.65	-0.5577	0.0491
12	Q15651	HMGN3	10.70	116.95	79.45	-0.5578	0.0489
13	P62310	LSM3	11.80	111.30	75.6	-0.5580	0.0486
14	P29401	TKT	67.80	105.20	71.45	-0.5581	0.0484
15	P15018	LIF	22.01	100.09	67.9	-0.5598	0.0483
16	P84243	H3-3A	15.32	134.35	91.1	-0.5605	0.0472
17	Q9UK76	JPT1	16.01	108.80	73.75	-0.5610	0.0468
18	Q96TA1	NIBAN2	84.10	109.15	73.95	-0.5617	0.0464
19	O14745	SLC9A3R1	38.80	109.80	74.35	-0.5625	0.0459
20	Q92945	KHSRP	73.1	111.30	75.35	-0.5628	0.0456

Annotations: “FC” =Fold change, “FDR”= False discovery rate
